# Feasibility study of direct CT lymphangiography in mice: comparison with interstitial CT/MR lymphangiography

**DOI:** 10.1007/s00330-023-09423-4

**Published:** 2023-01-31

**Authors:** Haruto Sugawara, Toshihiro Furuta, Akira Sumiyoshi, Megumi Iiyama, Masaru Kamitani, Aiko Suzuki, Arao Murakami, Osamu Abe, Ichio Aoki, Hiroyuki Akai

**Affiliations:** 1 Department of Radiology, Institute of Medical Science, University of Tokyo, 4-6-1 Shirokanedai, Minato-Ku, Tokyo, 108-8639 Japan; 2 Functional and Molecular Imaging Group, Department of Molecular Imaging and Theranostics, Institute for Quantum Medical Science, National Institutes for Quantum Science and Technology (QST), Chiba, 263-0004 Japan; 3 Department of Diagnostic Radiology, Kanto Rosai Hospital, Nakahara Ward, 1-1 Kizukisumiyoshicho, Kawasaki, Kanagawa 211-8510 Japan; 4 Department of Radiology, Graduate School of Medicine, University of Tokyo, 7-3-1 Hongo, Bunkyo-Ku, Tokyo, 113-8655 Japan

**Keywords:** Mice, X-ray microtomography, Lymphography, Contrast media

## Abstract

**Objectives:**

To establish a CT lymphangiography method in mice via direct lymph node puncture.

**Methods:**

We injected healthy mice (*n* = 8) with 50 µl of water-soluble iodine contrast agent (iomeprol; iodine concentration, 350 mg/mL) subcutaneously into the left-rear foot pad (interstitial injection) and 20 µl of the same contrast agent directly into the popliteal lymph node (direct puncture) 2 days later. Additionally, we performed interstitial MR lymphangiography on eight mice as a control group. We calculated the contrast ratio for each lymph node and visually assessed the depiction of lymph nodes and lymphatic vessels on a three-point scale.

**Results:**

The contrast ratios of 2-min post-injection images of sacral and lumbar–aortic lymph nodes were 20.7 ± 16.6 (average ± standard deviation) and 17.1 ± 12.0 in the direct puncture group, which were significantly higher than those detected in the CT or MR interstitial lymphangiography groups (average, 1.8–3.6; *p* = 0.008–0.019). The visual assessment scores for sacral lymph nodes, lumbar–aortic lymph nodes, and cisterna chyli were significantly better in the direct puncture group than in the CT interstitial injection group (*p* = 0.036, 0.009 and 0.001, respectively). The lymphatic vessels between these structures were significantly better scored in direct puncture group than in the CT or MR interstitial lymphangiography groups at 2 min after injection (all *p* ≤ 0.05).

**Conclusions:**

In CT lymphangiography in mice, the direct lymph node puncture provides a better delineation of the lymphatic pathways than the CT/MR interstitial injection method.

**Key Points:**

**•** *The contrast ratios of 2-min post-injection images in the direct CT lymphangiography group were significantly higher than those of CT/MR interstitial lymphangiography groups*.

**•** *The visibility of lymphatic vessels in subjective analysis in the direct CT lymphangiography group was significantly better in the direct puncture group than in the CT/MR interstitial lymphangiography groups*.

**•** *CT lymphangiography with direct lymph node puncture can provide excellent lymphatic delineation with contrast being maximum at 2 min after injection*.

## Introduction

Lymphatic leakage can be a fatal condition and requires early and appropriate treatment, and lymphangiography plays an important role in the success of interventional procedures for the lymphatic system [1]. In the past, lymphangiography has been performed by puncturing the lymphatic vessels in the dorsum of the foot [2]; however, direct puncture of the inguinal lymph nodes with subsequent embolization of the leakage site has become a widely accepted treatment in this context [3, 4]. Although lymphatic embolization has been mainly performed for chylothorax, there have been attempts to apply it to the treatment of chylous ascites as well [5, 6]. In any case, the use of appropriate contrast media and obtaining a clear lymphangiography are of vital importance for the success of this procedure.

In clinical practice, CT lymphangiography can be performed using X-ray positive-contrast agents to obtain more detailed images [7]. CT lymphangiography has some drawbacks such as radiation exposure, but it has some advantages over MR lymphangiography. First, CT has a better time and space resolution. This nature is particularly advantageous when the precise identification of an anatomical leakage point is necessary. Second, the subsequent interventional procedure is much easier because lymphatic interventions are often performed by puncturing enhanced structures, such as cisterna chyli, under fluoroscopy or CT guidance. The subsequent intervention can be performed immediately after CT lymphangiography when it is performed in the angio-CT suite, which is becoming increasingly popular. Third, ultrasound can be used in the scanning room. Lymphangiography by direct lymph node puncture is performed under ultrasound guidance in clinical practice. In MR lymphangiography, the patient must be transferred to the MR suite after puncturing the lymph node outside the room due to the restriction imposed by the magnetic field. CT has no such restriction, and imaging can be immediately performed after the ultrasound-guided lymph node puncture in the CT suite [8]. This would be a particularly important factor in cases where patient body movement is likely due to the unstable condition. Additionally, some patients have MRI contraindications because of the presence of metallic objects in their bodies, and CT lymphangiography could be their only option.

Basic studies of CT lymphangiography in animals are scarce. CT lymphangiography in swine [9], rabbits [10], and dogs [11] has been reported, but the application of this technique in mice, which are commonly used in fundamental laboratory experiments and are less expensive, has not been reported. By contrast, various reports on magnetic resonance (MR) lymphangiography in mice have been available in the literature since the early 2000s. A contrast agent is typically injected into the interstitium in mice during MR lymphangiography, and adjacent lymphatic structures have been reportedly visualized [12, 13]. This was possible because of the high contrast resolution of MRI. The contrast in CT lymphangiography via an interstitial injection is expected to be much weaker than that in MR lymphangiography with interstitial injection, considering the lower contrast resolution of CT. CT lymphangiography is projected to become more prevalent because of the increasing demand for lymphatic system–related interventional procedures. Therefore, conducting basic studies on the optimal amount, timing, and injection method of contrast medium using small experimental animals is highly desirable.

We examined the feasibility of CT lymphangiography in mice using direct lymph node puncture, and compared its image quality with that obtained via CT or MR interstitial lymphangiography.

## Materials and methods

All animal experiments were conducted in accordance with institutional guidelines. The experiments were approved by the institutions’ animal research committees (approval numbers: PA21-18 and 14–1006).

### Animals

Eight female BALB/c mice were purchased and maintained in a specific pathogen-free facility. All mice weighed approximately 20 g and had access to food and water ad libitum.

### CT lymphangiography

All images were obtained using a micro-CT for small animals (CosmoScan FX, Rigaku). The mice were anesthetized with 4% isoflurane in air. During imaging, 1.5% isoflurane was used. Before injection of the contrast medium, the mice were fixed in a mouse holder in a prone position and baseline images were acquired. The scanning parameters were as follows: tube voltage, 90 kV; tube current, 88 µA; exposure time, 18 s; field of view, 46 × 46 mm; and pixel size, 0.09 × 0.09 mm. After the acquisition of baseline images, 50 µl of a water-soluble iodine contrast agent (iomeprol; iodine concentration, 350 mg/mL) was slowly injected subcutaneously into the left-rear foot pad of the animals, and a gentle massage was applied for 30 s [14]. A previous case series of CT lymphangiography in humans reported that the thoracic duct was depicted 2–27 min after the start of contrast injection [7]. Additionally, a report of CT lymphangiography in swine with water-soluble contrast media shows the peak contrast at 5–10 min [8], and the contrast material starts to be washed out at a relatively early time point. The contrast was predicted to peak at an earlier timepoint because mice are smaller in size and can metabolize more quickly than humans or swine. Therefore, the imaging was performed under the same conditions at 2, 5, 10, 20, and 30 min after injection. At each time point, the range from the abdomen to the lower extremities was scanned first and the chest to the abdomen immediately thereafter.

**Fig. 1:**
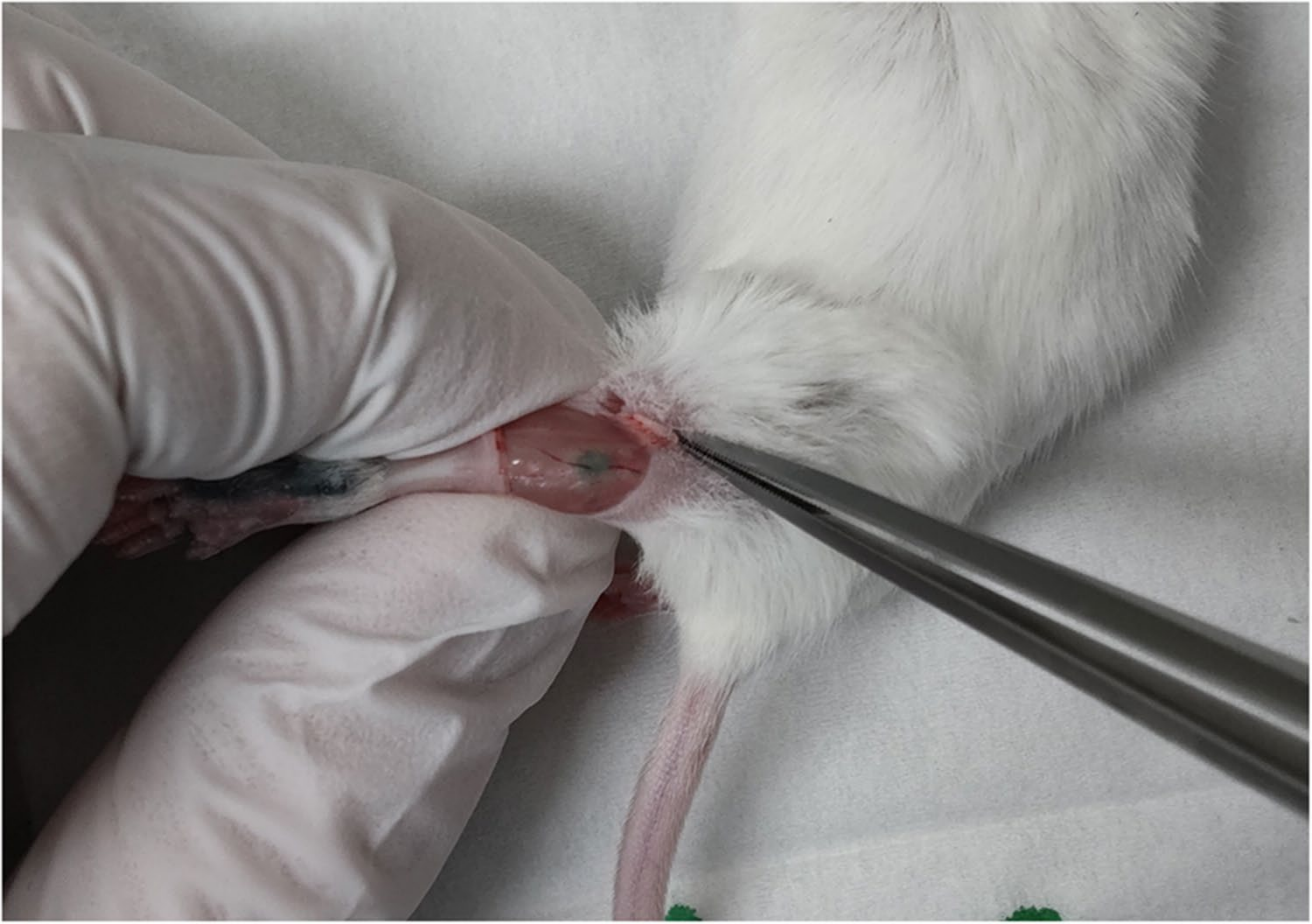
Popliteal lymph node of a mouse. After injection of indocyanine green into the rear foot pad, it becomes visible by incising the skin of the popliteal fossa

Two days later, 20 µl of 25 mg/mL indocyanine green was injected subcutaneously into the left-rear foot pad, and baseline CT images of the same mice were acquired using the same scanning parameters. Subsequently, the skin of the popliteal fossa was incised, to visualize the popliteal lymph nodes (Fig. 1), which were punctured directly and slowly injected with 20 µl of the iodine contrast material (iomeprol; iodine concentration, 350 mg/mL). CT images were obtained at 2, 5, 10, 20, and 30 min after injection.

### MR lymphangiography

As a control group, we performed interstitial MR lymphangiography on eight female BALB/c mice. The images were acquired using a 1-T MRI system (ICON, Bruker). The mice were anesthetized with 4% isoflurane in air and maintained with 1.5% isoflurane during the imaging procedure. The mice were taped to the mouse holder tightly in the prone position to mitigate motion artifacts. Before the contrast material injection, we obtained baseline 3D gradient echo T1-weighted images (TR, 40 ms; TE, 2.2 ms; flip angle, 45°; matrix, 256 × 128; bandwidth, 195 Hz/pixel; number of excitations, 1; slice thickness, 0.234 mm; 84 slices) in the coronal plane. We slowly injected diluted gadoteridol (Prohance, Bracco; 20 µmol in total) subcutaneously into the left-rear foot pad, and a gentle massage was applied for 30 s. The same 3D gradient echo T1-weighted imaging was repeated at 2, 5, 10, 20, and 30 min after injection.

### Image analysis

The time course of the obtained CT and MR lymphangiography images was quantitatively analyzed. A circular region of interest (ROI) was placed over the sacral and lumbar–aortic lymph nodes in the baseline image, and the average density or signal intensity inside the ROI was measured. Similarly, ROIs were placed on the sacral and lumbar–aortic lymph nodes in images obtained at 2, 5, 10, 20, and 30 min after contrast injection, and average internal densities or signal intensities were measured. The measured density or signal intensity of the lymph nodes in the baseline image was defined as *S*_pre_, and the density or signal intensity of the lymph nodes after contrast injection was defined as *S*_post_. The contrast ratio (CR) was calculated as follows:

$$\mathrm{CR}=\left({S}_{\mathrm{post}}-{S}_{\mathrm{pre}}\right)/{S}_{\mathrm{pre}}$$

**Fig. 2:**
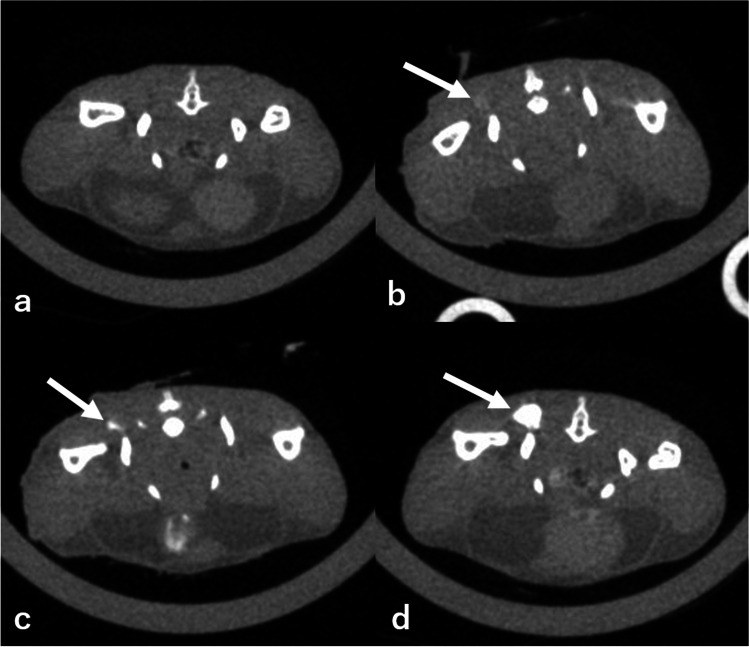
**a** Pre-contrast image, (**b**) an example of a poor score (minimal or no enhancement), (**c**) an example of a fair score (visible, but not strong enhancement), and (**d**) an example of a good score (strong enhancement). Arrows in the figure show the sacral lymph node

**Fig. 3:**
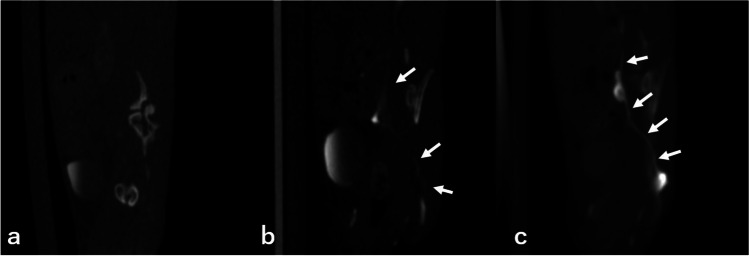
Coronal images of lymphatic vessels (mainly sacral are shown). **a** An example of a poor score, and almost no parts of lymphatic vessels are visible. (**b**) An example of a fair score. Although not as clear as (**c**), some parts of lymphatic vessels are visible. (**c**) An example of a good score. Most parts of lymphatic vessels are clearly visible

In the qualitative analysis, two radiologists (19 and 17 years of experience, respectively) evaluated the visibility of the sacral lymph node, lumbar–aortic lymph node, and cisterna chyli of axial images of 2 min after contrast injection. The visibility of the structure was categorized using the following 3-point scale based on a consensus between the two radiologists: good: strong enhancement; fair: visible, but not strong enhancement; and poor: minimal or no enhancement. Examples of each category are provided in Fig. 2. The lymphatic vessels between the popliteal and sacral lymph nodes, the sacral and lumbar–aortic lymph nodes, and the lumbar–aortic lymph nodes and the cisterna chyli were also evaluated. The visual score was graded on a three-point scale: good, almost the entire length of the lymphatic vessels is delineated; fair, lymphatic vessels are delineated approximately more than half of their total length; and poor, most of the lymphatic vessels are not delineated. We conducted only qualitative analysis for the lymphatic vessels because they are very small and difficult to quantitatively assess by placing ROIs. Examples of each category are provided in Fig. 3.

### Statistical analysis

The contrast ratio of sacral and lumbar–aortic lymph nodes at 2 min after injection in the direct puncture group was compared with that of the CT and MR interstitial injection group using *t* tests. For the qualitative study, the visual scores of lymph nodes and lymphatic vessels of the direct puncture group were compared with those of the CT and MR interstitial injection group using the Wilcoxon rank sum test. The statistical analyses were conducted using R software (ver 3.6.3), and significance was set at *p* ≤ 0.05.

## Results

**Fig. 4:**
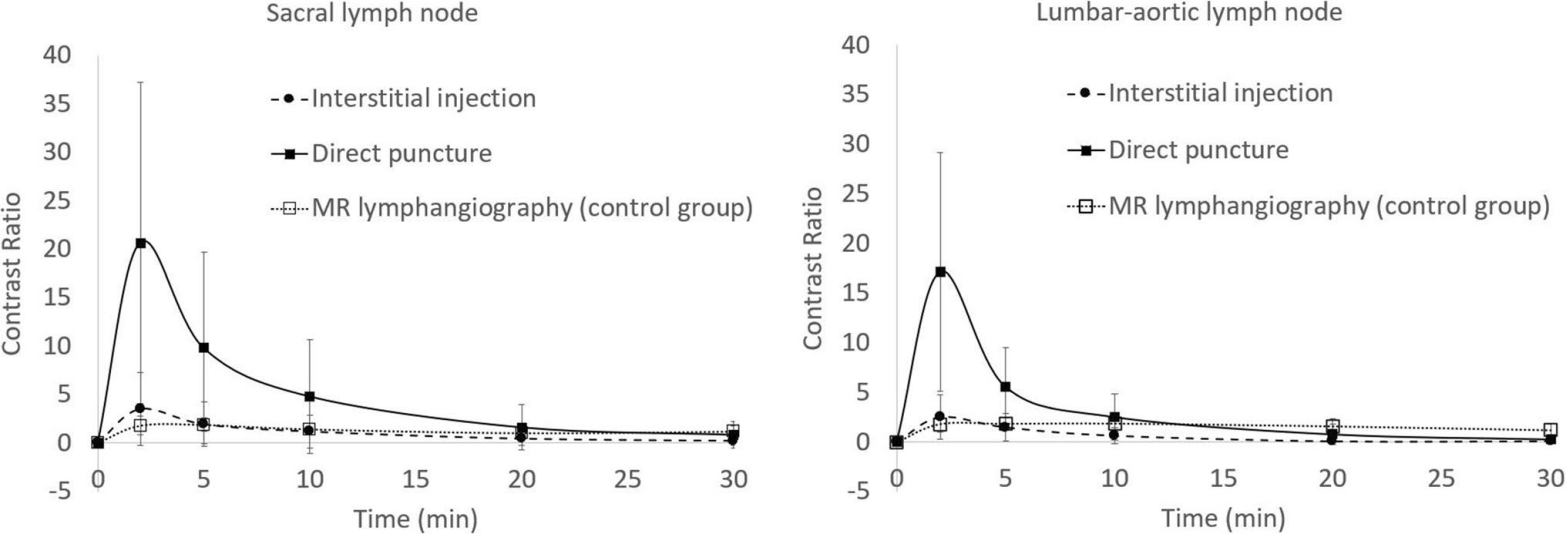
Time course of contrast ratio of sacral lymph nodes and lumbar–aortic lymph nodes. Both lymph nodes exhibited the highest contrast ratio at 2 min after injection; however, the peak was higher in the direct puncture group than it was in the CT/MR interstitial injection group. Bars represent mean ± standard deviation

Figure 4 shows the time course of the contrast ratio of sacral and lumbar–aortic lymph nodes after interstitial injection and direct puncture. In both injection methods, the maximum contrast ratio was observed in the images 2 min after injection on CT, but the peak was around 2–5 min after injection and the contrast remained relatively high throughout the period on MR. Regarding the sacral lymph nodes, their contrast ratio in the images acquired 2 min after injection was 20.7 ± 16.6 (average ± standard deviation) in the direct puncture group, which was significantly higher than the value of 3.6 ± 3.8 and 1.82 ± 0.93 recorded in the CT and MR interstitial injection group (*p* = 0.019 and 0.015, respectively). Similarly, for the lumbar–aortic lymph nodes, the contrast ratio in the images acquired 2 min after injection was 17.1 ± 12.0 in the direct puncture group, which was significantly higher than the value of 2.5 ± 2.3 and 1.82 ± 0.83 recorded in the interstitial injection group (*p* = 0.010 and 0.008, respectively).

Table 1 shows the results of the qualitative analysis. The visual scores of the sacral lymph nodes, lumbar–aortic lymph nodes, and cisterna chyli were significantly higher in the direct puncture group than they were in the CT interstitial injection group (*p* = 0.036, 0.009, 0.001, respectively); however, there was no significant difference between the direct puncture group and the MR interstitial injection group (*p* = 0.171–0.706). In particular, as for the cisterna chyli, 6 out of 8 (75%) mice in the direct puncture group showed strong enhancement and no mice exhibited poor (minimal or no enhancement) visibility, whereas 6 out of 8 (75%) mice in the CT interstitial injection group showed minimal or no enhancement.

**Table 1:** Visual score distribution in the qualitative assessment of lymph nodes 2 min after injection

	Interstitial injection (CT)	Interstitial injection (MR)	Direct puncture (CT)
Sacral lymph nodes	2/2/4	6/2/0	8/0/0
*p* value (vs direct puncture)	0.007^*^	0.467	
Lumbar–aortic lymph nodes	0/5/3	4/3/1	5/3/0
*p* value (vs direct puncture)	0.009^*^	0.706	
Cisterna chyli	0/2/6	3/3/2	6/2/0
*p* value (vs direct puncture)	0.001^*^	0.171	

For each cell of the table, the number of mice scored as being good/fair/poor is listed

^*^Statistically significant

Table 2 shows the results of the qualitative assessment of the lymphatic vessels. The lymphatic vessels between the popliteal and sacral lymph nodes, the sacral and lumbar–aortic lymph nodes, and the lumbar–aortic lymph nodes and the cisterna chyli were significantly better scored among the direct puncture group than the CT and MR interstitial group in the images 2 min after contrast injection (all *p* ≤ 0.05), whereas a significant difference was observed only in the lymphatic vessels between the popliteal and sacral lymph nodes in the images 10 min after injection.

**Table 2:** Visual score distribution in the qualitative assessment of lymphatic vessels 2 and 10 min after injection

	Interstitial injection (CT)	Interstitial injection (MR)	Direct puncture (CT)
2 min after injection			
Popliteal–sacral	0/2/6	0/2/6	7/1/0
*p* value (vs direct puncture)	< 0.001^*^	< 0.001^*^	
Sacral–lumbar–aortic	0/0/8	0/1/7	8/0/0
*p* value (vs direct puncture)	< 0.001^*^	< 0.001^*^	
Lumbar–aortic–cisterna chyli	0/1/7	0/0/8	4/3/1
*p* value (vs direct puncture)	0.005^*^	0.001^*^	
10 min after injection			
Popliteal–sacral	0/0/8	0/0/8	0/5/3
*p* value (vs direct puncture)	0.026^*^	0.026^*^	
Sacral–lumbar–aortic	0/0/8	0/0/8	0/3/5
*p* value (vs direct puncture)	0.2	0.2	
Lumbar–aortic–cisterna chyli	0/0/8	0/0/8	0/1/7
*p* value (vs direct puncture)	1.0	1.0	

For each cell of the table, the number of mice scored as being good/fair/poor is listed

^*^Statistically significant

When comparing images at 2 and 10 min after injection, the visual scores of the sacral–popliteal and popliteal–lumbar–aortic lymphatic vessels were significantly better in the direct puncture group at 2 min (*p* < 0.001). However, there were no significant differences in the lumbar–aortic–cisterna chyli lymphatic vessels in the direct puncture group and all lymphatic vessels in the interstitial puncture group.

**Fig. 5:**
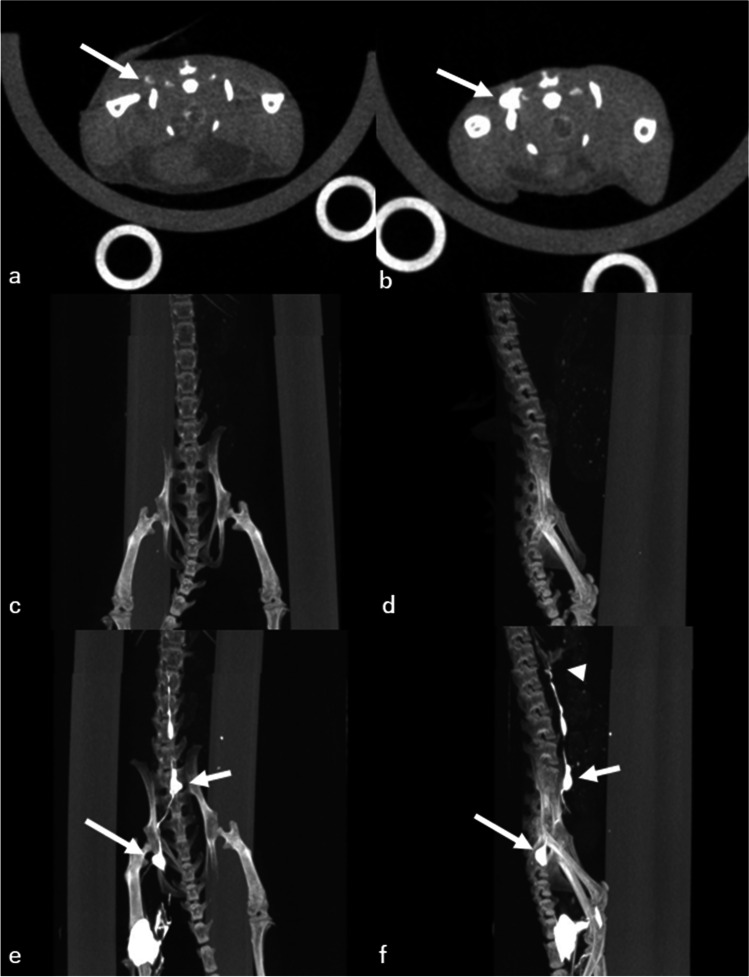
CT lymphangiography via interstitial injection (**a**) in a mouse, and CT lymphangiography via direct lymph node puncture (**b**) in the same mouse. The sacral lymph nodes (long arrow) were more strongly enhanced by direct lymph node puncture. **c** and **d** show the maximum intensity projection (MIP) of CT lymphangiography with interstitial contrast injection, whereas **e** and **f** show the MIP image of lymphangiography CT via direct lymph node puncture. It can be observed that CT lymphangiography with direct lymph node puncture depicted the lymphatic pathway (long arrow; sacral lymph nodes, short arrow; lumbar–aortic lymph nodes, arrowhead; cisterna chyli) more clearly. The MIP images were created by concatenating a part of the images obtained from the chest scan and the images obtained from the abdominal scan

Figure 5 shows the image that presents the difference in the lymphatic pathway visibility in CT lymphangiography between the direct puncture and interstitial injection methods. The contrast effect of the lymph nodes is weaker with interstitial injection than with direct puncture in axial CT images (Fig. 5a, b), as the quantitative and qualitative results suggest. This trend is even clearer when maximum intensity projection (MIP) images are created. The lymph nodes, nodules, and lymphatic vessels are almost invisible in the interstitial injection (Fig. 5c, d), whereas these structures and their interrelationships, as well as the entire lymphatic system, are clearly visualized in the direct puncture group (Fig. 5e, f).

## Discussion

This study revealed that CT lymphangiography is feasible using micro CT even in small experimental animals, such as mice. The injection of contrast material via direct lymph node puncture yields a stronger contrast effect using a lower amount of contrast agent compared with contrast injection into the interstitium, and lymphatic vessels were only delineated with direct lymph node puncture. The peak contrast was obtained 2 min after injection and the contrast decreased after 5 min.

The injection of contrast medium into the rear foot pad has been reported to provide feasible lymphatic enhancement in MR lymphangiography in mice [12, 13]. However, our study revealed that the interstitial injection into the foot pad can be insufficient for CT lymphangiography in mice, especially to depict lymphatic pathways that are distant from the injection site. With the interstitial injection, the cisterna chyli, which is the most distant structure from the puncture site, was not graded as “good” in any case, whereas with a direct puncture, not only lymph nodes relatively close to the puncture site but also the cisterna chyli were graded as well delineated by qualitative assessment. This provides strong evidence that accurate puncture of lymph nodes is imperative to obtain feasible images in CT lymphangiography using X-ray contrast agents in humans.

A similar trend can be observed for lymphatic vessel delineation. The present study revealed that most of the lymphatic vessels were rated as “poor” with interstitial injection, whereas most of them were rated as “good” with direct puncture. The lymphatic vessels are very small structures, and the difference in contrast between the interstitial injection and the direct puncture methods may have been particularly influential. Because both interstitial injection and direct puncture resulted in poorer lymphatic vessel depiction 10 min after injection compared to 2 min, the peak of lymphatic vessel delineation is also likely to occur approximately 2 min after injection.

In clinical practice, there have been reports that feasible MR lymphangiography was obtained by direct puncture of lymph nodes followed by injection of the gadolinium contrast agent [15, 16]. However, in humans, direct lymph node puncture is normally performed under ultrasound guidance, and the puncture using ultrasound should be performed outside the MR suite. As a solution to this problem, a previous study reported the feasibility of contrast-enhanced MR lymphangiography by subcutaneously injecting the gadolinium contrast agent between the toes [17]. CT lymphangiography with interstitial contrast injection was not reported in clinical practice and is not a routine method, but our study results suggest that sufficient visualization of important structures, such as lymphatic vessels and cisterna chyli, could not be obtained in CT lymphangiography with interstitial injection, unlike MR lymphangiography. Therefore, the direct lymph node puncture method would increasingly strengthen its position as the standard CT lymphangiography method. This would be because the concentration–signal curve of T1 contrast media is different from that of X-ray contrast media, such as iodine.

CT lymphangiography using a water-soluble iodine contrast agent has been reported recently [7]; however, that was a retrospective study in which images were obtained during actual clinical practice. Ethiodized oil (Lipiodol, Guerbet) is commonly used for lymphangiography in clinical practice, but the amount that can be used is limited due to the risk of embolizing non-target organs when entering the systemic circulation. In addition, it may not be suitable for dynamic studies due to their tendency to remain in the lymphatic vessels for a long period of time. Because of the relatively young history of CT lymphangiography with water-soluble contrast agent, many aspects need to be investigated to obtain improved images, such as the timing of imaging and the amount of contrast agent used. In the present study, the maximum contrast ratio in both sacral and lumbar–aortic lymph nodes was detected at 2 min after injection, and then gradually decreased after 5 min, and this was the main reason why we conducted visual assessment on images obtained 2 min after injection. In this study, obtaining images before 2 min post-injection was not practical because the mice had to be injected outside the micro-CT gantry due to the limited gantry space and then fixed on the mouse holder of the micro-CT system; the time point of the contrast peak may be even earlier than 2 min after injection. It would be preferable to perform CT lymphangiography as early as possible after the injection of the water-soluble contrast agent, to achieve the highest possible image contrast.

Water-soluble iodine contrast agents are widely used in clinical practice; however, pre-clinically, some X-ray positive-contrast agents, such as hepatocyte-selective iodinated triglyceride contrast agent [18] and nanoparticle-based non-iodinated contrast agents which shows higher contrast [19], have been reported to behave differently from water-soluble iodine contrast agents. Although these agents are not immediately available for use in clinical practice, they have the potential to dramatically improve the image quality of CT lymphangiography. Further investigations of CT lymphangiography using various contrast agents would be desirable.

Our study had several limitations. This is a preliminary study to confirm the feasibility of CT lymphangiography with the direct lymph node puncture method in mice. Therefore, the type and amount of contrast media have not been optimized. Further studies are needed to determine the optimal type and amount of contrast agent for CT lymphangiography. In addition, we performed CT lymphangiography using normal mice, rather that disease model mice, because our primary aim was to establish the basic CT lymphangiography technique.

In conclusion, we have demonstrated that CT lymphangiography was feasible in mice, and that CT lymphangiography via direct lymph node puncture yielded better images than the interstitial CT/MR lymphangiography.

## Abbreviations

CR: Contrast ratio
CT: Computed tomography
MR: Magnetic resonance
ROI: Region of interest

## Declarations

### Guarantor

The scientific guarantor of this publication is Hiroyuki Akai.

### Statistics and biometry

No complex statistical methods were necessary for this paper.

### Methodology

• experimental study
